# Hypoxia-Induced Alpha-Globin Expression in Syncytiotrophoblasts Mimics the Pattern Observed in Preeclamptic Placentas

**DOI:** 10.3390/ijms22073357

**Published:** 2021-03-25

**Authors:** Zahra Masoumi, Lena Erlandsson, Eva Hansson, Mattias Magnusson, Eva Mezey, Stefan R. Hansson

**Affiliations:** 1 Department of Clinical Sciences Lund, Division of Obstetrics and Gynecology, Lund University, SE-22184 Lund, Sweden; lena.erlandsson@med.lu.se (L.E.); eva.hansson@med.lu.se (E.H.); stefan.hansson@med.lu.se (S.R.H.); 2 Department of Molecular Medicine and Gene Therapy, Lund University, SE-22184 Lund, Sweden; mattias.magnusson@med.lu.se; 3 Adult Stem Cell Section, National Institute of Dental and Craniofacial Research, National Institutes of Health, 9000 Rockville Pike, Bethesda, MD 20892, USA; mezeye@nidcr.nih.gov; 4 Skåne University Hospital, SE-22184 Lund, Sweden

**Keywords:** preeclampsia, placenta, erythropoiesis, oxidative stress, non-erythroid globin, syncytiotrophoblast

## Abstract

Preeclampsia (PE) is a pregnancy disorder associated with placental dysfunction and elevated fetal hemoglobin (HbF). Early in pregnancy the placenta harbors hematopoietic stem and progenitor cells (HSPCs) and is an extramedullary source of erythropoiesis. However, globin expression is not unique to erythroid cells and can be triggered by hypoxia. To investigate the role of the placenta in increasing globin levels previously reported in PE, flow cytometry, histological and immunostaining and in situ analyses were used on placenta samples and ex vivo explant cultures. Our results indicated that in PE pregnancies, placental HSPC homing and erythropoiesis were not affected. Non-erythroid alpha-globin mRNA and protein, but not gamma-globin, were detected in syncytiotrophoblasts and stroma of PE placenta samples. Similarly, alpha-globin protein and mRNA were upregulated in normal placenta explants cultured in hypoxia. The upregulation was independent of HIF1 and NRF2, the two main candidates of globin transcription in non-erythroid cells. Our study is the first to demonstrate alpha-globin mRNA expression in syncytiotrophoblasts in PE, induced by hypoxia. However, gamma-globin was only expressed in erythrocytes. We conclude that alpha-globin, but not HbF, is expressed in placental syncytiotrophoblasts in PE and may contribute to the pathology of the disease.

## 1. Introduction

Preeclampsia (PE) is a pregnancy-related disorder affecting 3–8% of pregnancies worldwide [1]. Diagnosis of PE is based on maternal hypertension and evidence of organ damage presenting after 20 weeks of gestation [2]. The exact underlying mechanism of PE remains unknown, but placental dysfunction is central in the etiology. The disease starts at early stages of implantation with impaired invasion of trophoblasts into the decidua and inadequate remodeling of the spiral arteries [3]. This leads to impaired utero-placental circulation and uneven placental perfusion leading to intermittent hypoxia, which changes the cellular metabolism and induces oxidative stress [4,5]. These events lead to placental cell senescence and placental barrier damage, resulting in leakage of several factors into the maternal circulation [4,6]. Previous work in our lab has detected high levels of free fetal hemoglobin (HbF) in the maternal plasma [7] and local production of HbF in the placenta in PE [8], suggesting it may be one of the factors leaking into the maternal circulation. Free hemoglobin (Hb) and its toxic metabolites can cause oxidative stress, inflammation, and general endothelial damage leading to vasoconstriction and hypertension, renal failure, as well as thrombosis [9,10,11,12]. In fact, total plasma Hb level has been shown to correlate with systolic blood pressure in PE pregnancies [7].

The function of the placenta is crucial during fetal development as it provides the fetus with growth hormones and a means of gas and nutrition exchange [13]. During the first trimester, the placenta is also a significant extramedullary source of erythropoiesis and harbors a pool of hematopoietic stem and progenitor cells (HSPCs) [14,15,16,17,18]. There are limited data on how development, differentiation, and frequency of placental HSPCs may be affected by the inflammatory changes in the placental niche in PE [19]. It is also unclear whether these changes alter the migration and homing capacity of placental HSPCs, leading to prolonged placental erythropoiesis and local higher HbF production in PE [8].

The dogma that Hb production is unique to erythroid cells has been challenged over the years. Many studies have reported non-erythroid production of globin chains in macrophages, endothelial cells, and cancerous tumors, suggesting that globin expression is not specific to erythroid cells [20,21,22]. In the absence of the main erythroid-specific transcription factor of globin chains, GATA binding protein 1 (GATA1), other transcription factors such as hypoxia-inducible factor 1 (HIF1) and nuclear factor erythroid 2-related factor 2 (NFE2L2/ NRF2) have been suggested to regulate non-erythroid globin expression [23,24]. Both HIF1 and NRF2 are active and crucial for cell survival under hypoxic conditions and in oxidative stress [25,26,27]. Among the genes regulated by HIF1 and NRF2 transcription factors are heme oxygenase 1 (HMOX1) and the alpha- and beta-globin loci [28,29,30,31]. Remarkably, increased levels of both HMOX1 and HbF (comprised of alpha- and gamma-globin) have been detected in PE [8,32,33,34].

The aim of this study was to investigate potential placental contribution to higher HbF levels previously reported in PE. To investigate if erythropoiesis was prolonged in PE placentas, we explored the homing capacity of placental HSPCs (CD34^+^ CD45^+^ cells) by investigating their expression of surface adhesion molecules (SAMs) and by analyzing placental biopsies for production of erythroid precursors. Potential non-erythroid Hb expression was also compared between normotensive and PE placenta biopsies, as well as in placental explants from normotensive pregnancies cultured under normoxic or hypoxic conditions.

## 2. Results

### 2.1. Expression of SAMs on Placental HSPCs Was Not Significantly Different in PE

**Figure 1:**
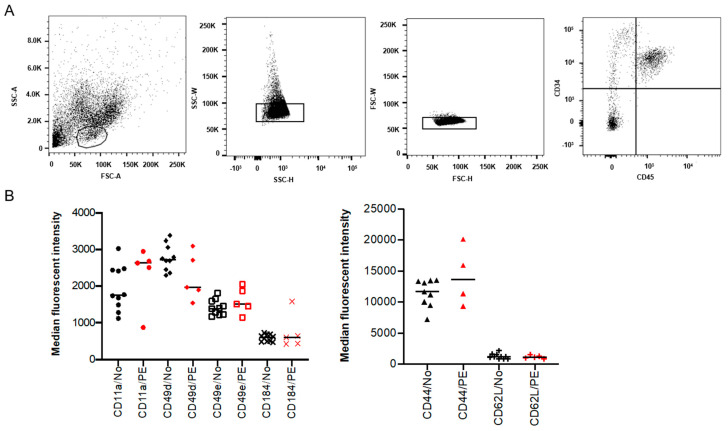
Surface adhesion molecules (SAMs) in isolated placental hematopoietic stem and progenitor cells (HSPCs) from preeclampsia (PE) and normotensive (NO) pregnancies. Flow cytometry analysis showing the isolated placental HSPC population gated based on size and granularity (FSC-A and SSC-A), and CD34^+^ CD45^+^ expression (**A**). Demonstrating the percentage of positive cells and median fluorescent intensity (MFI) for various SAMs in black (normotensive) and in red (PE) (**B**). The expression is not significantly different between PE (*n* = 5) and normotensive (*n* = 10).

To investigate if the increased frequency of placental HSPCs previously reported in PE pregnancies [18] is due to altered homing of the HSPCs, the expression of known SAMs was analyzed on isolated placental CD34^+^ CD45^+^ cells (Figure 1A). The expression of CD44, CD49d, CD49e, CD184 (CXCR4), CD11a and CD62L (L-selectin) was evaluated using flow cytometry. However, no significant differences were observed in the expression of these adhesion molecules between PE and normotensive pregnancies (Figure 1B).

### 2.2. Normotensive and PE Placentas Displayed Similar Distribution of Erythroid Cells and No Sign of Active Placental Erythropoiesis

**Figure 2:**
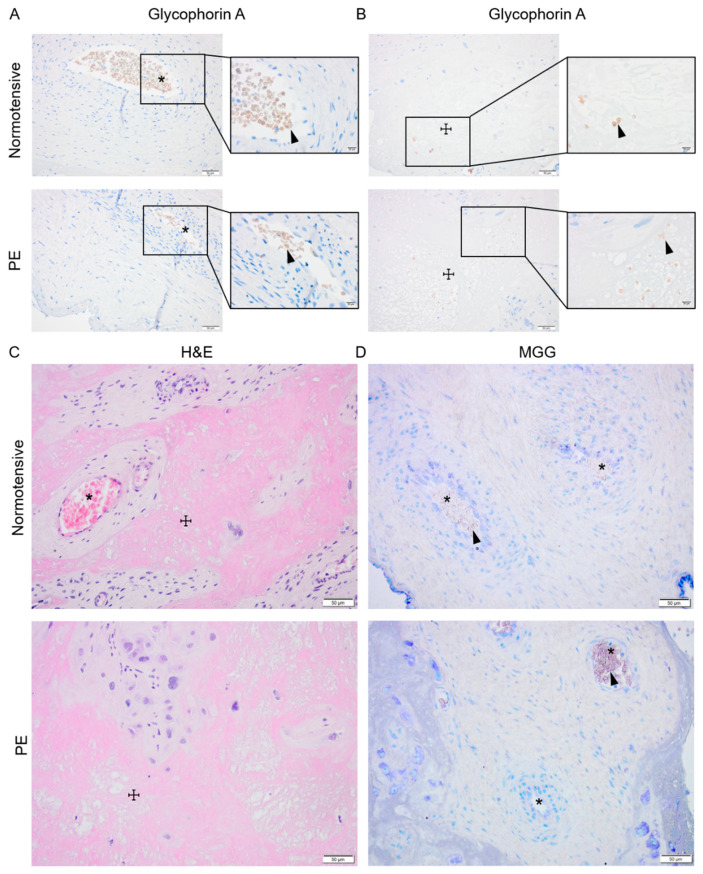
Investigating placental erythropoiesis and distribution of the erythroid cells in the placenta. Immunostaining for glycophorin-A (brown) indicating erythrocytes (arrowhead) in placental vessels (*) and areas with fibrosis (☩) in normotensive and PE placentas (**A**,**B**), the nuclei (dark purple) were stained by hematoxylin. Hematoxylin and eosin (H&E) staining of normotensive and PE placentas indicating the fibrinoid areas (**C**). May–Grünwald–Giemsa (MGG) staining in normotensive and PE placentas (**D**). Scale bar is 50 µm in all images except for inserted higher magnifications in (**A**,**B**) where it is 20 µm.

Signs of active placental erythropoiesis were analyzed to investigate whether previously described higher HSPC frequency [19] may lead to prolonged placental erythropoiesis and contribute to the elevated HbF levels previously detected in placenta and maternal circulation in PE [8,32,33]. Immunohistochemical analysis of glycophorin A (GYPA) expression as well as hematoxylin and eosin (H&E) and May–Grünwald–Giemsa (MGG) staining were performed on normotensive and PE placenta biopsies paired for sex and gestational age (Figure 2). In both groups, the GYPA^+^ erythroid cells were located in the placental vessels (Figure 2A) or trapped in areas (Figure 2B) with fibrinoid necrosis visualized by H&E staining (Figure 2C). The MGG staining, routinely used for distinguishing various hematopoietic cells, showed no erythroid islands in the chorionic plate or the intermediate villi in either PE or normotensive placentas (Figure 2D).

### 2.3. Protein Expression of Alpha-Globin, HIF1-Alpha and NRF2, But Not Gamma-Globin, Was Elevated in Early- and Late-Onset PE Placentas

**Figure 3:**
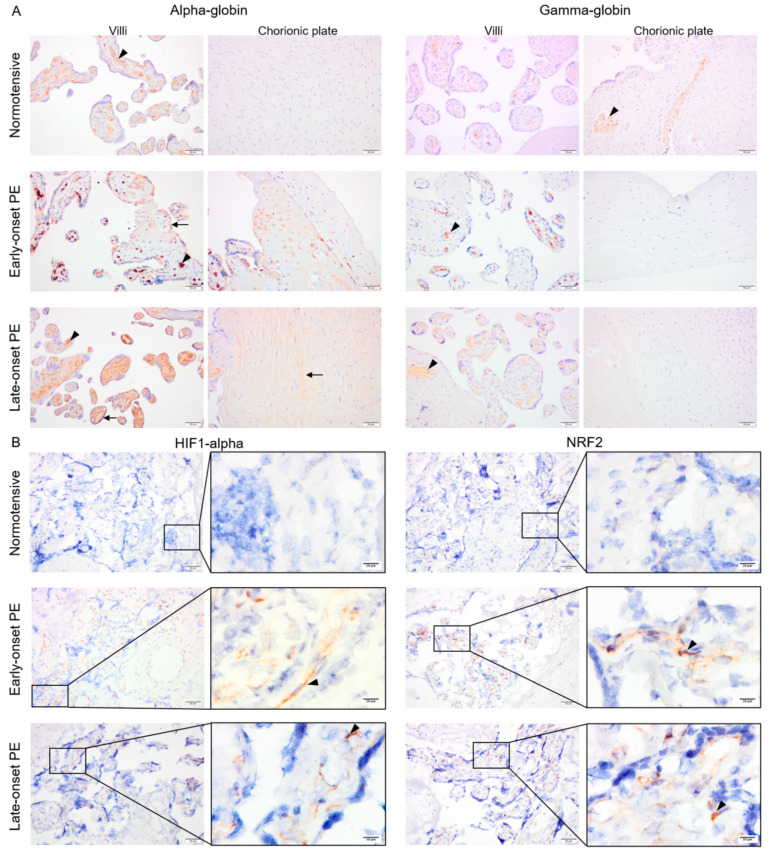
The pattern of alpha- and gamma-globin as well as HIF1-alpha and NRF2 proteins in placentas from normotensive, early-, and late-onset PE pregnancies. Alpha- and gamma-globin proteins were detected in erythrocytes in all three groups (arrowhead) (**A**). In early-onset PE, alpha globin protein was also clearly detected in trophoblasts and sparsely in the villous stroma (arrow) (**A**). In late-onset PE, alpha-globin was found in stroma of the villi and the chorionic plate (arrow) (**A**). Both HIF1-alpha and NRF2 protein expression were strongly detected in villous stroma of placentas from early- and late-onset PE pregnancies (arrowhead), but only minimal in normotensive placentas (**B**). Scale bar is 50 µm in all images except for inserted higher magnifications in B where it is 10 µm. Negative control is presented in Figure S1.

Fixed placenta samples from normotensive, early- and late-onset PE pregnancies were compared for alpha- and gamma-globin, HIF1-alpha as well as NRF2 protein expression. Alpha- and gamma-globin proteins were detected in erythrocytes inside vessels in all three study groups (Figure 3A). In addition, alpha-globin staining was also observed in trophoblasts and sparsely in stroma of the villi and the chorionic plate in early-onset PE and more homogenously in stroma of the villi and the chorionic plate in late-onset PE (Figure 3A), but not in normotensive placenta samples. Mean alpha-globin signal values from semi-quantification analysis in normotensive, early-, and late-onset PE placenta samples were 70.7, 107.8, and 100.8, respectively. The differences between normotensive and late- or early-onset PE groups were significant (*p* ≤ 0.005) but not between early- and late onset PE (*p* ≥ 0.4). Additionally, sex-specific alpha-globin expression was compared. The mean alpha-globin signal values in normotensive group were 73.7 in male and 68.7 in female placentas. These values increased to 113.8 for male and 92.1 for female placentas from late-onset PE group. However, no significant differences were observed in alpha-globin signal values between male vs. female groups in either normotensive or late-onset PE (*p* ≥ 0.5).

Both HIF1-alpha and NRF2 were detected within the villous stroma in early- and late-onset PE, which contrasted with normotensive placenta samples showing minimal staining (Figure 3B). After semi-quantification analysis, mean HIF1-alpha signal values were 14.9, 47.9, and 42.1 for normotensive, early-, and late-onset PE placenta samples, respectively. Mean NRF2 signal was 23.7, 52.7, and 47.4 in normotensive, early-, and late-onset PE groups, respectively. For both HIF1-alpha and NRF2, the differences were significant between normotensive samples and early- or late-onset PE (*p* ≤ 0.01) but not between early- and late-onset PE (*p* ≥ 0.4). 

### 2.4. Pattern of Alpha- and Gamma-Globin, HIF1-Alpha and NRF2 Protein Expression in Cultured Placental Explants

**Figure 4:**
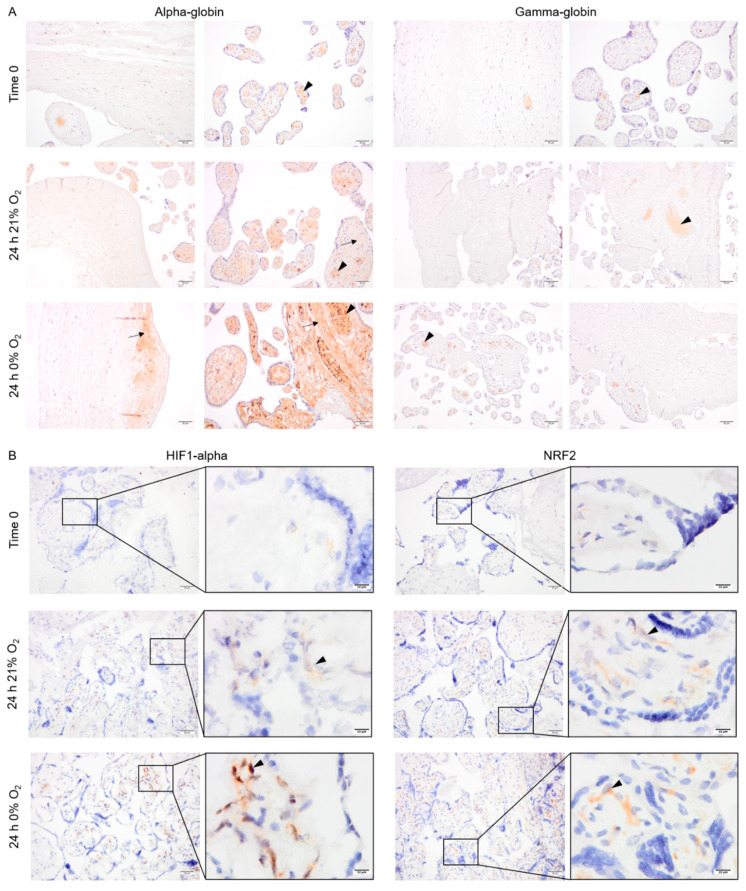
The pattern of expression of alpha- and gamma-globin as well as HIF1-alpha and NRF2 protein in placenta explants from normotensive pregnancies cultured in 0% or 21% oxygen. Alpha- and gamma-globin proteins were detected in erythrocytes (arrowhead) within the placenta vessels of explants cultured in 0% or 21% oxygen. Alpha-globin was also detected in villous stroma, particularly at 0% oxygen (arrow) (**A**). HIF1-alpha and NRF2 protein expression in placenta explants were detected in villous stroma, at both 0% and 21% oxygen levels (arrowhead). Scale bar is 50 µm in all images except for inserted higher magnifications in (**B**) where it is 10 µm. Negative control is presented in Figure S1.

Explants from normotensive placentas were cultured in 21% or 0% oxygen conditions. At time zero, the majority of alpha- and gamma-globin proteins were detected in erythrocytes inside the vessels (Figure 4A). After 24 h in culture, alpha-globin was detected in multiple cell types in placenta explants cultured at both 21% and 0% oxygen. The alpha-globin protein was mainly located in the villi, particularly in syncytiotrophoblasts covering the villi (Figure 4A). The gamma-globin, however, was only expressed by erythrocytes inside the vessels (Figure 4A). 

Interestingly, HIF1-alpha and NRF2 were stabilized and detected in the cytoplasm of several cell types within the villous stroma in the explants cultured for 24 h at either 0% or 21% oxygen (Figure 4B).

### 2.5. Non-Erythroid Transcription of Alpha-Globin Is Restricted to the Syncytiotrophoblasts in Hypoxia and PE

**Figure 5:**
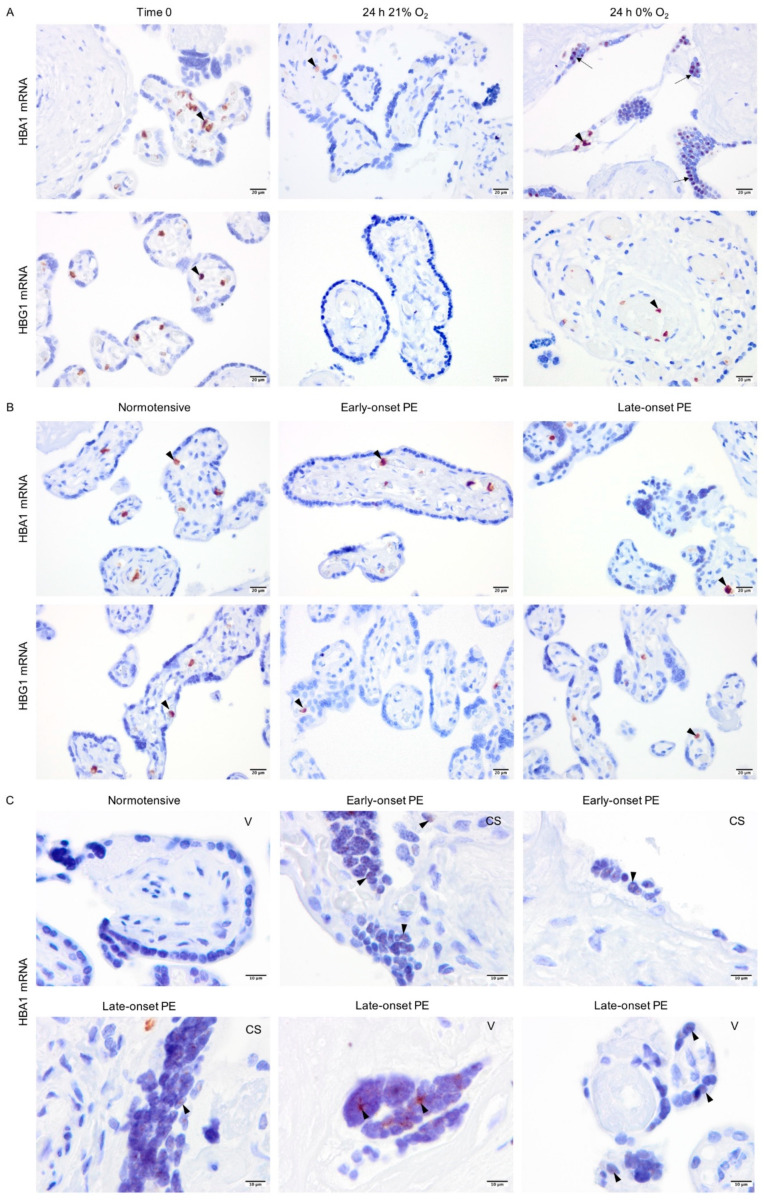
In situ analysis of alpha- and gamma-globin mRNAs. Alpha- and gamma-globin mRNAs were detected in erythrocytes in the placental vessels of cultured placental explants (arrowhead) (**A**). However, alpha-globin mRNA was also found in syncytiotrophoblasts of placenta explants cultured at 0% oxygen (arrow) (**A**). Alpha- and gamma-globin mRNAs were found in erythrocytes (arrowhead) in the placental vessels of placentas from both normotensive and PE pregnancies (**B**). Alpha-globin mRNA was also found sparsely in syncytiotrophoblasts (arrowhead) of placenta samples from early- and late-onset PE pregnancies independent of the mode of delivery; CS (cesarean section) or V (vaginal delivery) (**C**). Scale bar is 20 µm for A and B, and 10 µm for C. Images of negative control and placenta stroma are presented in Figures S1C and S2, respectively.

To localize the globin transcripts and to conclude any roles for HIF1-alpha or NRF2 transcription factors, *in situ* hybridization analyses were performed for alpha- (HBA1) and gamma-globin (HBG1) mRNAs. The HBG1 transcripts were only detected in erythrocytes localized in blood vessels in all explant cultures (Figure 5A). However, in line with alpha-globin protein expression, HBA1 mRNA was observed not only in erythrocytes but also sparsely in syncytiotrophoblasts covering the villi in placenta explants cultured at 0% oxygen (Figure 5A). In placenta samples from normotensive, early-, and late-onset PE pregnancies, both HBA1 and HBG1 mRNAs were observed in erythrocytes (Figure 5B). However, HBA1 mRNA was additionally detected in syncytiotrophoblasts covering the placental villi in early- and late-onset PE but not in the normotensive group (Figure 5C). The HBA1 expression pattern in PE placenta samples was also sparse and in line with the hypoxia-triggered alpha-globin transcription pattern observed in explant cultures (24 h 0% O_2_, Figure 5A). The HBA1-expressing syncytiotrophoblasts were present in PE placentas from both vaginal and cesarean section deliveries. 

## 3. Discussion

Altered placenta pathophysiology plays a central role in the development of PE. It is also suggested to be associated with elevated HbF levels [8] and increased frequency of HSPCs in the placental chorionic plate, which may lead to extended erythropoiesis [19]. In this study, we investigated erythroid and non-erythroid globin expression in the placenta to explore its potential contribution to the increased globin levels reported in PE. 

Placental HSPCs are generated de novo in the chorionic plate [17]. Surface adhesion molecules play a significant role in the migration, homing, and retention of HSPCs [35,36]. By analyzing the expression of SAMs, we investigated whether the intrinsic migration and homing capacity of placental HSPCs was altered in PE. Our results showed no significant differences in the expression of SAMs on the surface of placental HSPCs between PE and normotensive groups. While including more patients would provide higher statistical power to compare the groups, these data suggest that PE does not affect the intrinsic migration and homing capacity of placental HSPCs. Accordingly, the reported higher HSPC frequency in the chorionic plate in PE [19] may instead be due to changes in the placental niche, such as increased expression of SAM ligands, (e.g., hyaluronic acid [37]) that could potentially trap the HSPCs. 

It is established that the placenta is an extramedullary source of erythropoiesis, particularly during the first trimester of pregnancy [14,15,16,17,18]. We investigated changes in placental erythropoiesis as a source of the elevated HbF previously detected in PE. Erythroid cells were detected in the placental vasculature and within areas with fibrosis in both normotensive and PE placentas. However, no active erythropoiesis was observed in either group. This is in line with other studies limiting placental erythropoiesis to the first and early second trimester of pregnancy [18]. Our results suggest that placental erythropoiesis is not extended into late pregnancy in PE and therefore is unlikely to contribute to elevation of HbF previously described in PE [8,32].

Detection of globin chains in various cell types [20,21,22] suggest that Hb production is not limited to erythrocytes. Furthermore, HIF1 and NRF2 have been suggested to regulate transcription of globin genes in non-erythroid cells [23,24]. Hypoxia can affect cellular iron homeostasis and increase cellular heme deposits [38]. Free heme has cytotoxic and pro-oxidative effects [9,39], but it can also regulate the expression of antioxidant response element-controlled genes such as HMOX1 [40] and the globin loci [29,31,41]. It is considered that PE is associated with intermittent placental hypoxia [42,43], as well as altered levels of HMOX1 and HbF [8,32,33,34]. Hence, in the present study, we used monoclonal antibodies and specific probes to explore if hypoxia induced the expression of alpha- and gamma-globin chains in non-erythroid cells in the placenta. Our results showed that both gamma-globin mRNA and protein were strictly expressed in erythrocytes in all groups and culture conditions. The difference between the previously detected overproduction of HbF in PE using a polyclonal antibody [8] and the present gamma-globin localization in the placenta may be due to higher specificity of the antibody used in this study. Alpha-globin was similarly present in erythrocytes in all groups and cultures, but it was also present in non-erythroid cells, particularly in syncytiotrophoblasts. In general, alpha-globin protein was detected in placental stroma and in syncytiotrophoblasts in the explants cultured at 0% oxygen and in PE placenta samples compared to controls. Semi-quantification analyses indicated a significant increase in alpha-globin protein expression in both early- and late-onset PE samples while the differences between these two groups were not significant. Alpha-globin mRNA, on the other hand, was only expressed in the syncytiotrophoblasts in explants cultured in hypoxia; a pattern that was also observed in early- and late-onset PE placenta samples. These results suggest that hypoxia may alter the regulation of alpha-globin expression in syncytiotrophoblasts in PE. Mode of delivery has been shown to affect placental expression of several genes involved in regulation of oxidative stress, inflammation or metabolism [44]. However, alpha-globin was detected in PE placentas independent of the mode of delivery (cesarean section or vaginal), suggesting PE rather than delivery method affects the expression. Additionally, there have been reports describing patchy mRNA and protein expression patterns in trophoblasts [45,46,47], which is in line with the pattern of alpha-globin mRNA expression observed in syncytiotrophoblasts in this study. Lack of complete co-localization between alpha-globin mRNA and protein expression suggests that alpha-globin protein may be transferred from the syncytiotrophoblast to other proximal stromal cells. Intercellular transfer of cytoplasmic components, such as proteins, is an established means of cell communication [48,49,50]. Whether intercellular transfer of alpha-globin protein between placental cells is a means of communication or is designed to combat local hypoxia or oxidative stress requires further investigation. Globin expression in non-erythroid cells has been implied to decrease cellular oxidative stress [51] and to regulate iron metabolism [52], mitochondrial hemostasis [53], as well as nitric oxide signaling [21]. Alpha-globin expression in endothelial cells has been shown to regulate arterial vascular reactivity by controlling endothelial nitric oxide synthase signaling and nitric oxide diffusion to smooth muscle cells at the myoendothelial junction [21]. Interestingly, placental syncytiotrophoblast-derived vesicles are released into the maternal circulation during pregnancy and can be taken up by vascular endothelial cells [54], which may point to a mechanism underlying maternal hypertension in PE. 

Fetal sex is an important factor that determines maternal adaptation to pregnancy [55], as well as the onset of the disease and severity of maternal symptoms in PE [56,57]. The placenta function and structure [58] as well as fetal and placental metabolism [59] are also regulated by fetal sex. Thus, potential sex-specific differences in alpha-globin expression were analyzed in male vs. female placentas in both normotensive and late-onset PE groups. However, no significant differences were observed between male and female placentas, although including more patients may provide higher statistical power to compare the groups. Additionally, while hormones such as testosterone have been suggested to control globin expression in erythroid cells [60] and play a significant role in PE [61,62,63], lack of a significant difference in alpha-globin expression in male vs. female placentas may be due to distinct globin transcription regulations in non-erythroid cells. 

To better understand the regulation of hypoxia-induced alpha-globin transcription in non-erythroid cells, known candidate transcription factors, HIF1 and NRF2, were analyzed [23,24]. The mechanisms underlying stabilization of NRF2 and HIF1-alpha in the cytoplasm and their nuclear translocation following hypoxia-induced oxidative stress are well known [25,26,27]. In the nucleus, HIF1-alpha binds HIF1-beta subunit to make up the active HIF1 transcription factor. Activation of HIF1 and NRF2 is associated with transcription of several genes and induction of survival pathways in hypoxia [25,26,27]. However, some studies have reported that stabilization and activation of HIF1 and NRF2 can also occur in response to oxidative stress triggered by hyperoxia [64,65]. This may explain the detection of HIF1-alpha and NRF2 in placenta explants cultured at 21% oxygen. Both HIF1-alpha and NRF2 were detected at significantly higher levels in early- and late-onset PE vs. normotensive placenta samples. However, neither HIF1 nor NRF2 were localized to the syncytiotrophoblast nuclei in either placenta explants or samples, indicating that other transcription factors may control the expression of alpha-globin in syncytiotrophoblasts. 

## 4. Materials and Methods

### 4.1. Ethical Approval

The study was approved by the Ethics Committee Review Board for studies on human subjects at Lund University and Skåne University Hospital, Lund, Sweden (Dnr 2014/191). After receiving written informed consent from the patients, placentas from normotensive and PE pregnancies were collected following both cesarean section and vaginal deliveries at Skåne University Hospital. Preeclampsia was defined as blood pressure ≥140/90 mmHg and proteinuria ≥300 mg/L [2]. Early- or late-onset PE were defined as delivery earlier or later than 34 gestational weeks, respectively. The placentas were stored at 4 °C and processed within 4 h after delivery. A summary of the clinical demographics of the patients included in this study and the subsequent experiments are listed in Table S1. 

### 4.2. Placenta Tissue Digestion and Isolation of Mononuclear CD34^+^ Cells

To isolate mononuclear CD34^+^ cells, placentas from 10 normotensive and 5 late-onset PE pregnancies were used (Table S1). An average of 85 g of tissue was collected per placenta, including the chorionic plate but avoiding the cotyledons and villi. The tissue was cut into small pieces using scissors and washed in a total of 1 L of 1× phosphate-buffered saline (PBS) (Gibco^®^, Thermo Fisher Scientific, Waltham, MA USA). These pieces were mechanically digested in PBS in C tubes (Miltenyi Biotec, Bergisch Gladbach, Germany) for 2 min using the “h-skin-01” program on a gentleMACS^™^ Dissociator (Miltenyi Biotec, Bergisch Gladbach, Germany). The tissue was further digested enzymatically in 400 mL of digestion buffer containing 1 mg/mL collagenase II (Sigma Aldrich, Saint Louis, MO, USA), 250 μg/mL dispase II (Gibco^®^, Thermo Fisher Scientific, Waltham, MA USA), 10 μg/mL DNase I (Sigma Aldrich, Saint Louis, MO, USA), 100 IU/mL penicillin, 100 μg/mL streptomycin (both Gibco^®^, Thermo Fisher Scientific, Waltham, MA USA), 10 μg/mL ciprofloxacin (Sigma Aldrich, Saint Louis, MO, USA), and 2.5 μg/mL amphotericin B (Sigma Aldrich, Saint Louis, MO, USA ), on a shaker at 200 rpm for 45 min at 37 °C along with intermittent gentle pipetting. Digestion was terminated by the addition of 10% ice-cold fetal bovine serum (FBS) (Gibco^®^, Thermo Fisher Scientific, Waltham, MA USA) and single-cell suspension was obtained by filtering through 100 μm, 70 μm, and 40 μm filters, consecutively. Each filtration step was followed by centrifugation at 350× *g* at 4 °C and resuspending the cells in ice-cold wash buffer (5% FBS, 2 mM EDTA in PBS). Finally, total mononuclear cells (MNCs) were isolated using Ficoll-Paque PLUS (GE Healthcare Life Sciences, Danderyd, Sweden) according to the manufacturer’s protocol. In brief, the placental cell suspension was carefully laid upon Ficoll-Paque PLUS and centrifuged at 400× *g* at room temperature (RT) for 30 min. The interphase layer containing the MNCs was retrieved and thereafter mixed 1:2 with ice-cold Iscove’s Modified Dulbecco’s Medium with 10% FBS (Gibco^®^, Thermo Fisher Scientific, Waltham, MA USA). The cell suspension was centrifuged at 300× *g* for 10 min at 4 °C and the cell pellet was rinsed in wash buffer twice before further analysis.

The placental CD34^+^ cells were selected using the human CD34 MicroBead Kit (Miltenyi Biotec, Bergisch Gladbach, Germany) according to the manufacturer’s protocol. The MNCs were incubated with FcR Blocking Reagent and CD34 MicroBeads at 4 °C for 30 min, then rinsed and centrifuged at 300× *g* at 4 °C for 10 min. The cell suspension was passed through a 40 μm filter and CD34^+^ cells were separated using magnetic selection on LS columns (Miltenyi Biotec, Bergisch Gladbach, Germany). Cells were stained and analyzed for SAMs by flow cytometry using a BD FACSAria^TM^.

### 4.3. Flow Cytometry Analysis of SAM Expression on Placental HSPCs

Placental HSPCs were detected by flow cytometry as CD34^+^ CD45^+^ cells and the expression of 6 different SAMs was analyzed using phycoerythrin-conjugated mouse anti-human antibodies recognizing integrin α4 (Int α4)/CD49d, integrin α5 (Int α5)/CD49e, integrin αL (Int αL)/CD11a, chemokine (C-X-C motif) receptor 4 (CXCR4)/CD184, CD44, and L-selectin/CD62L (BD biosciences, San Jose, CA, USA) (Table 1) [36,66,67,68,69]. To perform the staining, six separate suspensions of CD34^+^-enriched cells were prepared and an appropriate amount of antibody (1:25) specific for CD34 (CD34-phycoerythrin/Cy7) and CD45 (CD45-FITC) in combination with one of the 6 SAMs was added to each preparation followed by 30 min incubation on ice. After rinsing the cells, they were analyzed using a BD FACSAria™ I. Spectral compensation was carried out using VersaComp Antibody Capture Beads kit (Beckman Coulter, Brea, CA, USA) and gates were set based on unstained as well as fluorescent minus one control. As a viability marker, 7-aminoactinomycin D (7AAD) was used at 10 μg/mL (Sigma Aldrich, Saint Louis, MO, USA). The data were analyzed by two-tailed Mann–Whitney U test using FlowJo (V.10.0.8).

### 4.4. Evaluation of Placental Erythropoiesis 

Placental erythropoiesis was assessed in a total of 12 normotensive and 12 late-onset PE samples paired for fetal sex and gestational age (Table S1). All procedures were performed at RT unless otherwise stated. The placenta tissue biopsies were cut from an area within a 7 cm radius of the umbilical cord, rinsed in PBS and fixed in 4% buffered formaldehyde solution (Histolab^®^, Västra Frölunda, Sweden) for 24 h before paraffin embedding and subsequent sectioning. The sections (4 μm) were deparaffinized in xylene, rehydrated in decreasing concentrations of ethanol before antigen retrieval in slow boiling 10 mM citrate buffer (pH = 6.0) for 20 min. To block endogenous peroxidase activity, sections were treated with 1% hydrogen peroxide for 10 min before blocking non-specific protein binding sites (Dako, X0909, Agilent, Santa Clara, CA, USA) for 20 min. To detect the erythroid cells, sections were incubated with mouse anti-human glycophorin A (GYPA) (1:50, BD Biosciences, San Jose, CA, USA) at 4 °C overnight, followed by horseradish peroxidase (HRP)-conjugated secondary polyclonal goat anti-mouse antibody (1:200, Dako, Agilent, Santa Clara, CA, USA) for 30 min. Detection was performed using DAB solution (Dako, Agilent, Santa Clara, CA, USA) according to manufacturer’s instructions followed by counterstaining with hematoxylin. In addition, May–Grünwald–Giemsa (MGG) [70] and hematoxylin and eosin (H&E) staining were performed to detect erythroid islands [71] and to analyze tissue morphology, respectively.

### 4.5. Placenta Explant Cultures

A total of 6 placentas were collected from normotensive pregnancies (Table S1). Placentas were sampled according to a systematic uniform random sampling method at 10 random sites [72]. Tissue samples (5 × 5 mm) were rinsed in PBS and a total of 200 mg explants were cultured in 35 mm culture dishes in 3 mL of Dulbecco’s Modified Eagle Medium supplemented with FBS (10%), penicillin (100 U/mL), and streptomycin (100 µg/mL) (all from Gibco^®^, Thermo Fisher Scientific, Waltham, MA USA) and cultured at 37 °C in 21 or 0% oxygen (the medium was not degassed prior to culture). After 24 h culture, the explants were quickly washed in PBS and fixed either by snap freezing on dry ice and stored at −80 °C or by submersion in 4% buffered formaldehyde solution (Histolab^®^, Västra Frölunda, Sweden) prior to paraffin embedding.

### 4.6. Immunostaining of HIF1-Alpha, NRF2, Alpha-, and Gamma-Globin 

Along with the placental explants, placenta samples were prepared from 12 normotensive, 12 late-onset, and 5 early-onset PE pregnancies (Table S1). Immunohistochemistry was performed for HIF1-alpha, NRF2, and the fetal Hb chains (alpha- and gamma-globin). Sections were prepared at 10-micrometer thickness from frozen OCT-embedded tissue and were fixed in ice-cold acetone for 10 min at −20 °C for HIF1-alpha and NRF2 staining. The paraffin-embedded sections were prepared and treated as mentioned above and antigen retrieval was performed using 10mM Tris-EDTA buffer (pH = 9.0) for alpha- or gamma-globin antibodies. Endogenous peroxidase activity and non-specific protein binding sites were blocked in all slides as mentioned earlier. The sections were then incubated with a primary antibody detecting HIF1-alpha (mouse anti-human, clone 54, BD Transduction lab, 1:100), NRF2 (mouse anti-human, clone A10, 1: 100 Santa Cruz Biotechnology, Inc., Santa Cruz, CA, USA), alpha-globin (Rabbit anti-human, Clone EPR3608, 1:250, Abcam, Cambridge, UK ), or gamma-globin (mouse anti-human, clone 51-7, 1:250, Santa Cruz Biotechnology, Inc., Santa Cruz, CA, USA) overnight at 4 °C. After 30-minute treatment with the appropriate HRP-conjugated secondary antibody (goat anti-rabbit (1:50, Dako, Agilent, Santa Clara, CA, USA) or goat anti-mouse (1:200, Dako, Agilent, Santa Clara, CA, USA), detection was performed using DAB solution (Dako, Agilent, Santa Clara, CA, USA) according to manufacturer’s instructions. Negative controls for each immunostaining were incubated with secondary antibody only. Nuclear counterstaining was performed using hematoxylin. The slides were dehydrated and mounted by PERTEX^®^ (Histolab^®^, Västra Frölunda, Sweden) prior to microscopy.

### 4.7. In Situ Hybridization Analysis of Alpha- and Gamma-Globin mRNAs 

To investigate gene transcription, in situ hybridization analysis of alpha-globin (HBA1) and gamma-globin (HBG1) mRNAs was performed using RNAScope^®^ technology (Advanced Cell Diagnostics (ACD)-Bio-Techne, Newark, CA, USA) according to the manufacturer’s protocol. In brief, sections (4 μm) were prepared from paraffin-embedded placenta tissues from 12 normotensive, 12 late-onset, and 5 early-onset PE pregnancies as well as 6 normotensive placenta explants cultured at 21 or 0% oxygen. (Table S1) After heating at 60 °C for 1 h, the slides were cooled to RT for 10 min, deparaffinized in xylene and rinsed in absolute ethanol before drying at RT. The endogenous peroxidase activity was blocked by 1% hydrogen peroxide treatment for 10 min at RT. The sections were quickly rinsed in dH_2_O and treated by target retrieval at 98 °C for 15 min. After a 15-second rinse in dH_2_O, the samples were incubated with absolute ethanol for 3 min at RT. The samples were dried at 60 °C and treated with protease reagent in a humid chamber at 40 °C for 30 min. After preparing the RNAScope^®^ probes for HBA1, HBG1, and RNAScope^®^ negative control, the slides were rinsed in 1X RNAScope^®^ Wash Buffer Reagent and hybridization was performed in a humid chamber at 40 °C for 2 h. After rinsing the slides, amplification of the signal was performed using AMP 1- AMP 6 from RNAScope^®^ HD detection reagents (Brown) kit. After the last amplification, appropriate mix of DAB (A)–DAB (B) solution was added to the slides for 10 min at RT. Negative controls incubated with Negative Control Probe-DapB were included for each in situ hybridization analysis. Counterstaining was carried out using 50% hematoxylin followed by a quick rinse in ammonium hydroxide solution (0.02%) (Sigma-Aldrich^®^). The slides were dehydrated prior to mounting by PERTEX^®^ (Histolab^®^, Västra Frölunda, Sweden) and microscopy.

### 4.8. Semi-Quantification of the Immunostaining 

To compare the staining of alpha-globin, HIF1-alpha, and NRF2 between groups, semi-quantification of the immunostaining analyses was performed. To cover maximum tissue variance and area while maintaining good signal-to-noise ratio, 60× objective was used to take 3 TIFF images per sample.

Using ImageJ, image segmentation was used to semi-quantify the DAB signal. Each image was opened in ImageJ and:

A. The image was deconvolved (Image: Color: Color Deconvolution: Vectors:
  H DAB) and the DAB (brown) layer of the image was saved. This image was inverted (Edit: Invert) so that background (white) would equal zero and signal (black) would equal 255 (Figure S3).
B. Then, the image was used to create a mask to select the area of interest following these steps:
  1. Image: Type: 8bit;
  2. Image: Adjust: Threshold (Setting: B&W, No Dark Background selection; Apply);
  3. Process: Binary: Fill holes;
  4. Analyze: Tools: ROI manager;
  5. Area selected by Wand (tracing) tool: add to ROI manager;
  6. To remove an area within the selection (i.e., removing vessels prior to measuring alpha-globin), the area was marked, added to ROI, and removed using the “XOR” command;
  7. An area of 50 × 50 µm was also selected on an area of background with no tissue present (Figure S3).

These selected areas were transferred to the inverted DAB layer (A) by choosing them on the ROI manager (Figure S3). The measurements of interest were set (Analyze: Set Measurements: area, Mean grey value). By choosing “Measure” on the ROI manager, the mean overall signal was measured for the selected areas. After deducting the background signal, the value was considered as the mean signal value for DAB staining for each image. Single factor ANOVA and two-sample t-test (assuming unequal variance) were used for further statistical analysis.

## 5. Conclusions

This study indicates that PE neither affects migration capacity of the placental HSPCs, nor leads to prolonged placental erythropoiesis in the third trimester of pregnancy. Thus, prolonged placental erythropoiesis does not contribute to HbF elevation previously described in PE. However, PE activates expression of alpha-globin in syncytiotrophoblasts, which is independent of mode of delivery. Data from the in vitro experiments support the notion that hypoxia may induce alpha-globin expression in syncytiotrophoblasts in PE. Further investigation is necessary to elucidate the potential role of alpha-globin in placental cells and in the etiology of PE. 

## Figures and Tables

**Table 1:** The markers used in flow cytometry and immunohistochemistry analyses.

Target	Markers/Profile
Hematopoietic stem/progenitor cells (HSPCs)	CD34+ (clone 581)
	CD45+ (clone HI30)
Surface adhesion molecules (SAMs)	CD44 (clone 515)
	CD49d (clone 9F10)
	CD49e (clone IIA1)
	CD184 (clone 12G5)
	CD11a (clone HI111)
	CD62L (polyclonal)

## Data Availability

Data is contained within the article and supplementary material.

## Supplementary Materials

The following are available online at https://www.mdpi.com/1422-0067/22/7/3357/s1, Figure S1: Negative controls for antibodies and in situ hybridization probes, Figure S2: In situ analysis of alpha- and gamma-globin mRNAs in placenta stroma, Figure S3: Semi-quantification of the images from immunostaining analyses using ImageJ, Table S1: Detailed description of clinical characteristics of the patients for each set of experiments [73].

## Abbreviations

FBS	Fetal bovine serum
GATA1	GATA-binding protein 1
GYPA	Glycophorin A
H&E	Hematoxylin and Eosin
HBA1	Alpha-globin 1
HbF	Fetal hemoglobin
HBG1	Gamma-globin 1
HIF1	hypoxia-inducible factor 1
HMOX1	Heme oxygenase 1
HSPC	Hematopoietic stem and progenitor cell
MNC	Mononuclear cell
MGG	May-Grunwald Giemsa
NRF2	Nuclear factor erythroid 2-related factor 2
PE	Preeclampsia
SAM	Surface adhesion molecule
