# Supplementary material for: Hypoxia-Induced Alpha-Globin Expression in Syncytiotrophoblasts Mimics the Pattern Observed in Preeclamptic Placentas
Source: Int J Mol Sci. 2021 Mar 25;22(7):3357. doi: 10.3390/ijms22073357 (PMC8036899; doi:10.3390/ijms22073357)
Supplement: Supplementary file 1 [file ijms-22-03357-s001.pdf]

Table S1: Detailed description of clinical characteristics of the patients for each set of experiments.

Pat#	Fetal sex	Fetal weight (g)	Gestational age (W+D)	Percentile ranks (Z score)	Growth Classification[*†]	Mode of delivery	Maternal age	Maternal BMI	Parity	Gravity	Pregnancy condition
**Samples used in placenta HSPC isolation and SAM expression analysis[‡]**											
P871	Male	3490	39+4	45 (-0.13)	AGA	Vaginal	35	26	2	2	Normotensive
P875	Male	4285	40+3	88 (1.22)	AGA	Cesarean section	35	21	1	1	Normotensive
P877	Female	4720	40+1	99.6 (2.65)	LGA	Vaginal	34	22	2	2	Normotensive
P879	Female	4600	42+1	94 (1.53)	LGA	Vaginal	35	22	2	2	Normotensive
P880	Female	3905	41+0	62 (0.47)	AGA	Vaginal	27	22	2	3	Normotensive
P882	Male	3755	41+3	37 (-0.31)	AGA	Vaginal	24	24	1	1	Normotensive
P884	Male	2345	36+3	94 (1.54)	LGA	Vaginal	30	25	2	2	Normotensive
P885	Female	3495	38+2	75 (0.71)	AGA	Vaginal	20	19	2	2	Normotensive
P886	Male	4420	39+1	99 (2.21)	LGA	Vaginal	24	24	1	1	Normotensive
P887	Female	4000	42+1	58 (0.29)	AGA	Vaginal	32	33	1	1	Normotensive
P876	Male	3790	39+2	74 (0.68)	AGA	Vaginal	31	22	2	3	Late-onset PE
P891	Male	3415	39+5	36 (-0.36)	AGA	Vaginal	29	29	2	3	Late-onset PE
P892	Female	1925	37+3	1 (-2.94)	SGA	Cesarean section	38	24	1	1	Late-onset PE
P894	Male	3805	36+1	99.7 (2.7)	LGA	Vaginal	27	22	1	2	Late-onset PE
P907	Female	4100	39+2	95 (1.66)	LGA	Vaginal	30	34	0	1	Late-onset PE

Samples used for erythropoiesis analysis (GYPA, MGG) and H&E staining**, immunostaining for HIF1-alpha, NRF2, alpha- and gamma-globin as well as *in-situ* analysis of alpha- and gamma-globin mRNA											
**Pat#**	**Fetal sex**	**Fetal weight (g)**	**Gestational age (W+D)**	**Percentile ranks (Z score)**	**Growth Classification*†**	**Mode of delivery**	**Maternal age**	**Maternal BMI**	**Parity**	**Gravity**	**Pregnancy condition**
P912	Male	4015	39+3	87 (1.12)	AGA	Vaginal	41	25	1	3	Normotensive
P914	Male	2905	36+4	47 (-0.08)	AGA	Vaginal	19	23	0	1	Normotensive
P945	Male	4195	40+5	82 (0.9)	AGA	Vaginal	34	22	1	2	Normotensive
P922	Male	3220	38+6	33 (-0.44)	AGA	Vaginal	30	17	0	2	Normotensive
P886	Male	4420	39+1	99 (2.21)	LGA	Vaginal	24	24	1	1	Normotensive
P956	Male	3600	39+5	52 (0.05)	AGA	Vaginal	29	20	1	2	Normotensive
P954	Male	3820	39+2	77 (0.75)	AGA	Vaginal	29	25	1	3	Late-onset PE
P951	Male	2950	36+0	65 (0.38)	AGA	Vaginal	36	24	1	2	Late-onset PE
P961	Male	4546	40+4	96 (1.71)	LGA	Vaginal	34	24	0	1	Late-onset PE
P899	Male	3155	38+5	30 (-0.53)	AGA	Vaginal	26	30	0	1	Late-onset PE
P930	Male	2574	37+0	12 (-1.19)	AGA	Vaginal	34	20	1	3	Late-onset PE
P973	Male	4740	39+6	99 (2.-52)	LGA	CS	27	24	0	1	Late-onset PE
P968	Female	3354	38+1	67 (0.43)	AGA	vaginal	27	NA	1	3	Normotensive
P911	Female	3160	39+6	22 (-0.77)	AGA	Vaginal	29	23	0	1	Normotensive
P947	Female	3880	40+0	79 (0.81)	AGA	Vaginal	22	23	0	1	Normotensive
P958	Female	3305	36+5	87 (1.11)	AGA	Vaginal	39	22	2	1	Normotensive
P928	Female	3935	39+1	91 (1.34)	LGA	Vaginal	35	22.6	0	1	Normotensive
P946	Female	3160	37+5	56 (0.15)	AGA	Vaginal	24	22	0	1	Normotensive
P893	Female	3140	38+4	38 (-0.3)	AGA	Cesarean section	33	29	1	3	Late-onset PE

P907	Female	4100	39+2	95 (1.66)	LGA	Vaginal	30	34	0	1	Late-onset PE
P963	Female	3900	40+0	81(0.86)	AGA	Vaginal	35	22	0	1	Late-onset PE
P974	Female	2440	36+6	9 (-1.34)	SGA	vaginal	30	NA	0	2	Late-onset PE
P959	Female	3215	39+3	32 (-0.48)	AGA	Vaginal	39	25	0	2	Late-onset PE
P892	Female	1925	37+3	0.1 (-2.94)	SGA	Cesarean section	38	24	1	1	Late-onset PE
P896	Male	1610	32+2	4 (-1.73)	SGA	Cesarean section	39	23	0	1	Early-onset PE
P932	Male	650	26+0	0.5 (-2.57)	SGA	Cesarean section	29	24	1	2	Early-onset PE
P936	Male	2520	34+6	43 (-0.18)	AGA	Cesarean section	21	24	0	1	Early-onset PE
P920	Female	1698	33+0	4 (-1.7)	SGA	Cesarean section	40	25	2	5	Early-onset PE
P923	Female	1085	30+1	0.4 (-2.61)	SGA	Cesarean section	21	33	0	1	Early-onset PE

**Samples used for placenta explant cultures**											
**Pat#**	**Fetal sex**	**Fetal weight (g)**	**Gestational age (W+D)**	**Percentile ranks (Z score)**	**Growth Classification[*†]**	**Mode of delivery**	**Maternal age**	**Maternal BMI**	**Parity**	**Gravity**	**Pregnancy condition**
P01	Male	2230/ 2590	36+3	3 (-1.86)/ 19 (-0.87)	SGA/AGA	Cesarean section	32	27	4	2	Normotensive
P02	Female	3830	39+1	6 (1.09)	SGA	Cesarean section	36	NA	0	1	Normotensive
P03	Male	3090	38+6	23 (-0.74)	AGA	Cesarean section	31	33	5	3	Normotensive
P04	Male	3085	39+3	16 (-0.99)	AGA	Cesarean section	29	22	0	2	Normotensive
P05	Male	3100	38+6	23 (-0.72)	AGA	Cesarean section	33	21	1	3	Normotensive
P06	Female	4345	38+2	99.7 (2.83)	LGA	Cesarean section	30	34	2	3	Normotensive

*Growth Classification was based on the Z score. Z score < -1.27 are small for gestational age (SGA), -1.27 < Z score < 1.27 are considered appropriate for gestational age (AGA), and Z score > 1.27 are large for gestational age (LGA).
**Erythropoiesis analysis and H&E staining (figure 1) were performed on placenta samples from normotensive and late-onset PE pregnancies.
†The LGA and SGA samples were analyzed for Doppler abnormalities. No abnormalities were observed except in case of P923 where uterine arterial score, blood flow class and pulsality index were 4, 1 and 1.54, respectively, suggesting fetal growth restriction.
‡These samples were previously used to analyze SAM expression on cord blood HSPCs [73].
NA: Not available

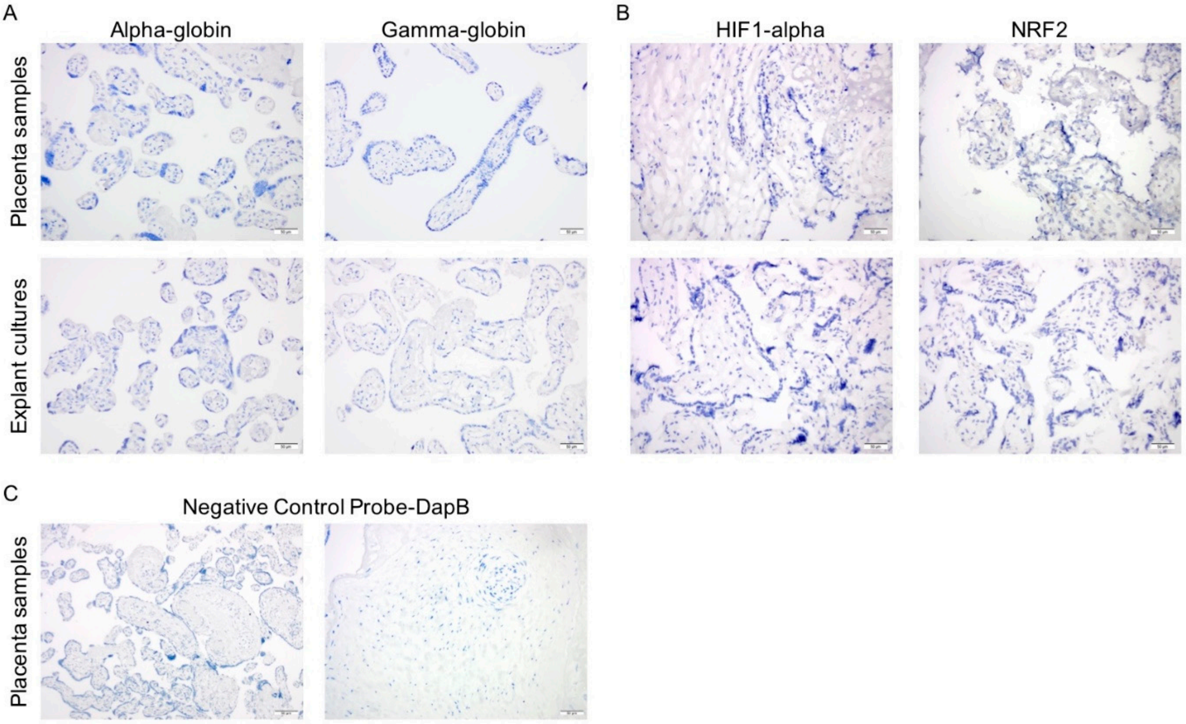

Figure S1. Negative controls for antibodies and *in si*tu hybridization probes. Negative controls for alpha- and gamma-globin antibodies used in Figure 3A (placenta) and in Figure 4A (cultures) (A) as well as for HIF1-alpha and NRF2 antibodies used in Figure 3B (placenta) and Figure 4B (cultures) (B). Negative control for HBA1, HBG1 *in situ* hybridization probes used in Figure 5 (C). The scale bar is 50 μm.

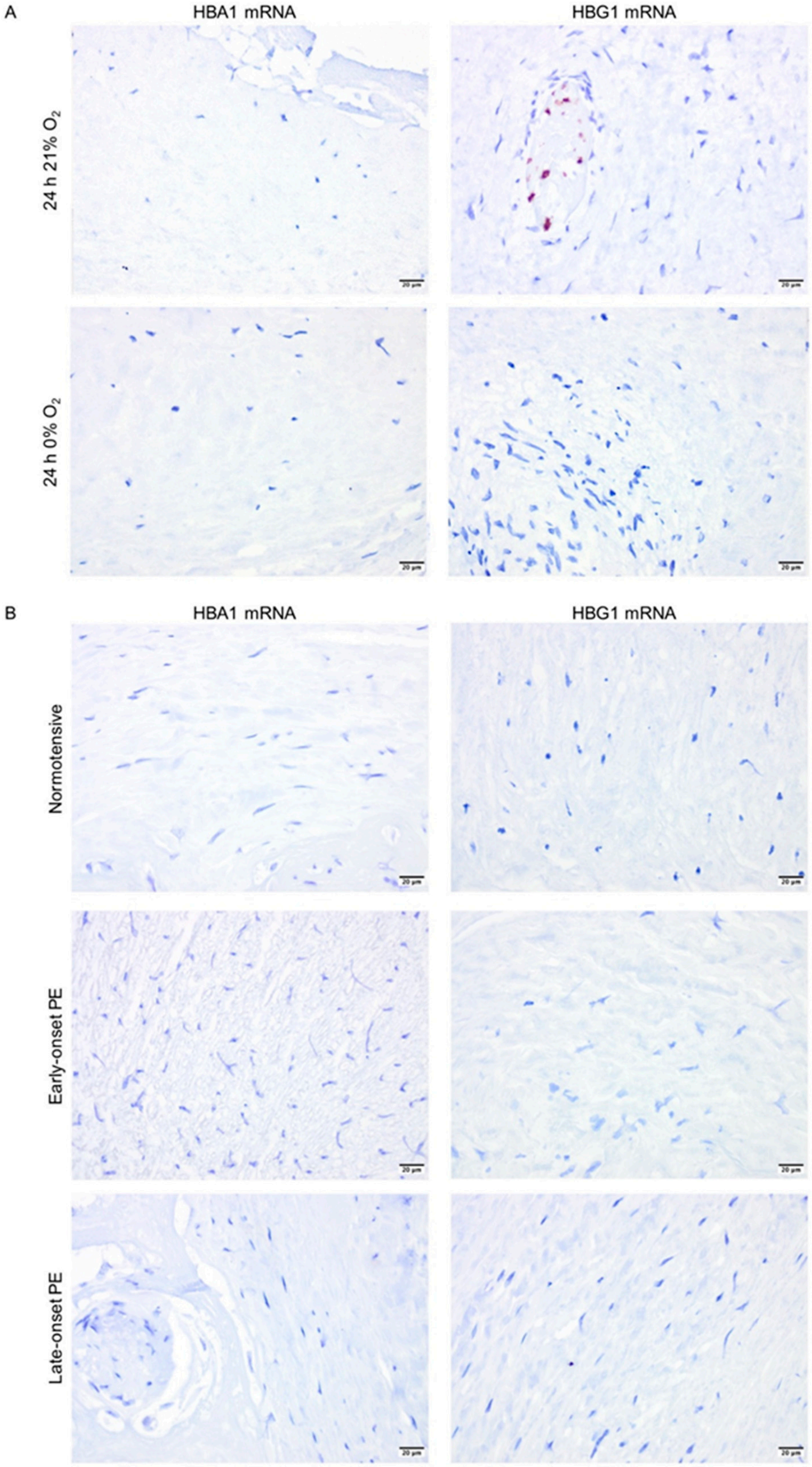

Figure S2. *In situ* analysis of alpha- and gamma-globin mRNAs in placenta stroma. Despite detection of alpha-globin protein, neither alpha- nor gamma-globin were detected in the placenta chorionic plate stroma in either control or explants cultured in hypoxia or PE samples. The scale bar is 20 μm.

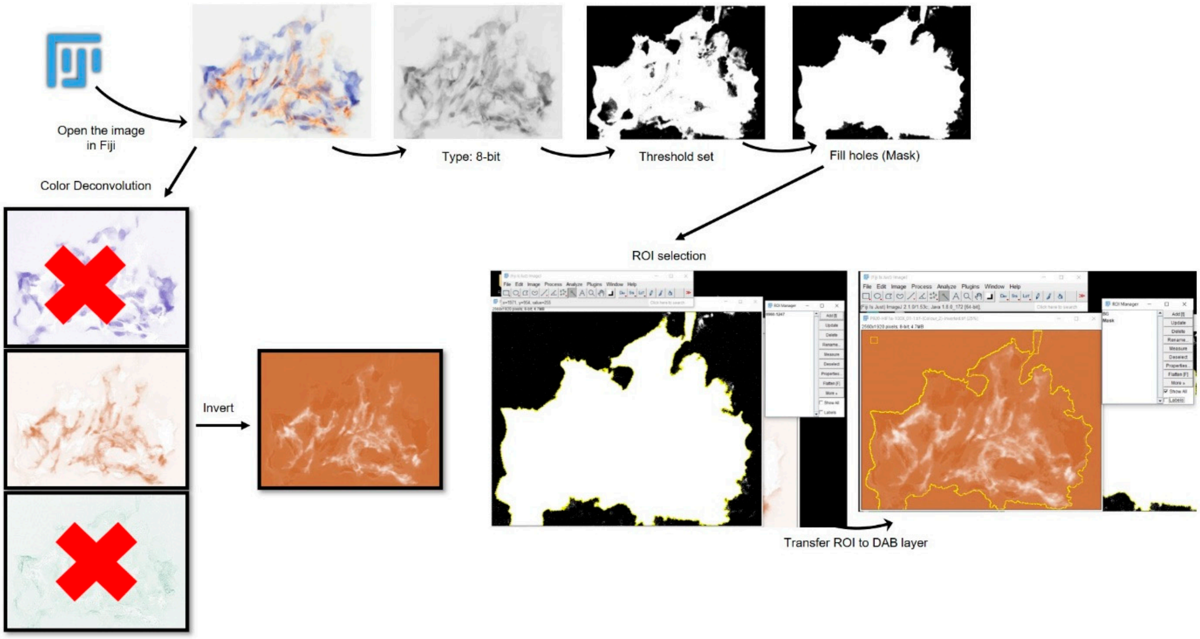

Figure S3. Semi-quantification of the images from immunostaining analyses using ImageJ.
